# Age of migration and common mental disorders among migrants in early adulthood: a Norwegian registry study

**DOI:** 10.1186/s12888-024-05963-1

**Published:** 2024-07-22

**Authors:** Melanie L. Straiton, Dawit Shawel Abebe, Lars Johan Hauge

**Affiliations:** 1 Division of Mental and Physical Health, Norwegian Institute of Public Health, P.O. Box 222, Skøyen, Oslo 0213 Norway; 2 Oslo Metropolitan University, P.O. Box 4, St Olavs plass, Oslo 0130 Norway; 3 Norwegian National Advisory Unit on Concurrent Substance Abuse and Mental Health Disorders, Innlandet Hospital Trust, P.O. Box 104, Brumunddal, NO-2381 Norway

**Keywords:** Migrants, Mental disorder, Health service use, Acculturative stress, Gender socialisation

## Abstract

**Background:**

Younger age of migration is associated with higher risk of psychotic disorders but the relationship between age of migration and common mental disorders is less clear. This study investigates the association between age of migration and diagnosed common mental disorders among migrants living in Norway.

**Methods:**

Using national Norwegian register data from 2008 to 2019, we compared the odds of a common mental disorder diagnosis in healthcare services during early adulthood among non-migrants, descendants and migrants with different ages of migration and lengths of stay. We also investigated differences in the relationship for different migrant groups and for men and women.

**Results:**

Descendants and childhood migrants with ≥ 19 years in Norway had higher odds of common mental disorders than non-migrants, while those migrating during adolescence with ≥ 19 years in Norway had similar odds. Those migrating during emerging and early adulthood had lower odds. Overall among migrants, the relationship between age of migration and common mental disorders was more pronounced for migrants < 19 years in Norway than ≥ 19 years and for non-refugees compared with refugees, especially men.

**Conclusions:**

Descendants and childhood migrants with long stays may have higher odds of common mental disorders due to the associated stress of growing up in a bicultural context compared with non-migrants. Age of migration has a negative association with diagnosed common mental disorders but much of this effect may attenuate over time. The effect appears weaker for refugees, and particularly refugee men, which may reflect higher levels of pre-migration trauma and stress associated with the asylum-seeking period for those arriving as adults. At the same time, migrants, especially those arriving as adults, experience barriers to care. This could also explain the particularly low odds of diagnosed common mental disorders among adult migrants, especially those with shorter stays.

**Supplementary Information:**

The online version contains supplementary material available at 10.1186/s12888-024-05963-1.

## Background

Migration is risk factor for mental disorder [1, 2]. The risk of mental disorder, however, varies according to a number of migratory-associated factors including reason for migration, country of origin, country of destination and length of stay [1–3]. Age of migration may also be an important factor. A systematic review and meta-analysis showed that the increased risk of psychotic disorders among migrants pertained mostly to individuals migrating as minors [4]. Those migrating during early adulthood had a similar risk of psychotic disorders as non-migrants, while those migrating before age 18 had twice the risk.

The association between age of migration and common mental disorders (CMDs) such as depression and anxiety however, is less clear [5–8]. It could partly be dependent on the life-stage CMDs are studied. In the current study, we investigate the relationship between age of migration, length of stay and CMDs diagnosed in healthcare services during early adulthood. Early adulthood appears to be an important period of risk for CMD onset (Kessler et al., 2007) and the prevalence is higher than in middle or late adulthood (Jacobi et al., 2015). Yet, most of the literature on age of migration and CMDs focuses on middle to late adulthood [9, 10] or adulthood in general [5–8, 11, 12].

Differences in findings may also relate to the study setting or country. For adulthood in general, a German study found no link between age of migration and mental health-related quality of life [7]. Researchers from Sweden, however, found that among migrants of working age, migrants arriving as children reported a lower risk of psychological distress than migrants arriving as adults, though the advantage was smaller among those with longer stays [5]. The authors argued that childhood migrants have more cultural exposure and opportunities for integration than migrants arriving as adults, which may be positive for their mental health. Indeed, research shows that the younger the age of arrival, the higher the likelihood an individual has of achieving higher education, employment and high income [13]. These factors are associated with better mental health [14–16]. At the same time, continued exposure to socioeconomic inequalities over time appeared to attenuate any positive effect of lower age of migration on mental health [5].

A negative relationship between age of migration and psychological distress, depressive symptoms and diagnosed CMDs during adulthood has, in contrast, been demonstrated in studies from the USA and Canada [6, 8, 11, 12, 17]. There are several explanations for why migrants arriving under the age of 18 may be at greater risk of CMDs during adulthood than those migrating as adults. Firstly, childhood and adolescence are considered sensitive developmental periods. Experiencing major stressors such as those associated with migration during these life phases can set the base for later negative health outcomes [18–20]. Children and adolescents are developing their independence, identity and finding their place within social groups. Uprooting during these periods can be particularly challenging. Difficulties in consolidating one’s identity can manifest in mental health problems during early adulthood [21].

Second, it is possible that the increased risk of CMDs is not due to the experience of migration itself, but rather related to the multiple interacting processes in the social, cultural and ecological context in which a child with a migrant background grows and develops [22]. Acculturation is an especially important process related to the social, emotional and cognitive development of children growing up in bi- and multicultural contexts [23]. The child’s sense of belonging to both their culture of origin and the dominant culture can lead to acculturative stress [24, 25]. Young migrants and descendants of migrants are also met with multiple disadvantages, such as growing up in low income households [26], social exclusion, discrimination and racism across the life course [27]. Being exposed to discrimination and social exclusion from a young age can also have an impact on futuremental health [28]. Beginning during a sensitive period and cumulating over time, these chronic stressors could result in an increased risk of CMDs during early adulthood compared with their non-migrant counterparts and migrants arriving as adults. Similarly, adolescents are working on consolidating their identity and exposure to discrimination and social exclusion can undermine this process, leading to increased risk of mental health problems [29].

A third argument for why a younger age of migration may increase the risk of CMDs relates to the healthy migrant effect. Those who choose to migrate are mostly young, have a higher level of education and better health status than non-migrants [30]. It is adults, and not children, who tend to drive migration, and the future health of a young child may not be a consideration for a healthy parent. Thus, we might expect migrants arriving as adults to have a lower risk of CMDs in early adulthood than migrants arriving as children. Further, giving the health migrant hypothesis, migrants arriving as adults might be less likely to experience CMDs than their non-migrant counterparts, while migrants arriving as children could have a similar risk.

However, research also suggests that the healthy migrant effect tends to diminish over time [31]. Migrants can experience many stress factors such as financial hardship and other socioeconomic inequalities, language difficulties, difficulties in understanding and navigating the society, discrimination and social exclusion [32, 33]. This cumulative stress may increase the risk of CMDs over time, also for adults. Thus, it is possible that any mental health advantage which migrants arriving as adults may experience compared to migrants arriving as children could diminish over time.

Yet, there is limited evidence of a healthy migrant effect when it comes to mental health [33], especially in Europe. It may also be dependent on country of origin and reason for migration [34, 35]. Although international refugees must be in good enough health to manage a long, and often difficult, journey, (adult) refugees have not chosen to migrate to the same extent and may therefore not be in as good health as other groups of (adult) migrants. Further, preparedness for migration may be important for future mental health in the settlement country [36]. Adults are likely to be more prepared for moving than children are. Due to the forced nature of migration for refugees, adult refugees may plan their migration and settlement to a lesser extent than non-refugee adult migrants, while refugee and non-refugee children may have similar levels of preparedness. Thus, if there is a negative relationship between age of migration and CMDs as a result of the healthy migrant effect, it could be weaker for refugees compared with non-refugees.

A final aspect to consider is the intersection between age of migration, gender and migrant group. Boys and girls may experience differences in socialisation, including family and peer relationships, and differences in exposure and responses to stress and social adversities (e.g., discrimination) [27, 37]. Girls, for instance, tend to experience more parental control and restrictions on their activities outside of the home [38], especially among those from lower income countries [37]. Limited freedom or negative social control is associated with poorer mental health [39]. US researchers found that Latinas who migrated during childhood were at higher risk of suicidal ideation than Latinos migrating during adulthood, while there was no difference for men and women among those migrating during adulthood [40]. They argued that Latina childhood migrants experience more gender oppression and family conflict than childhood Latino migrants, resulting in poorer mental health. In contrast to migrant girls, migrant women from countries with more conservative values will have already consolidated their identity prior to arrival, be more likely to have social ties with people from a similar background and may feel less pressure to conform to the expectations in the dominant society. Thus, they may experience lower levels of cultural conflict. As such, difference in CMDs between men and women among migrants from countries where gender differences in socialisation are greater (e.g. some countries outside of the European Economic Area (EEA), USA, Canada, Australia and New Zealand), could be larger for those arriving during childhood compared to those arriving during emerging or late adulthood.

In summary, there are several aspects of the association between age of migration and CMDs that still need to be investigated. European research is limited and contradicts literature from North America. This could be due to the different country contexts and migration policies (e.g. more selective migration in North America) or the dominant migrant groups in different countries. To our knowledge there are no Norwegian studies on this topic. Differences could also be due to the study design or quality of the data. Outcome measures in studies vary from self-reported psychological distress to depression diagnoses measured through diagnostic interviews. Most studies, however, rely on cross-sectional, self-reported data, which are subject to both selection and recall bias. Further, few studies consider whether migrants are at differential risk to individuals without a migrant background, depending on the age of migration [5, 7].

## Current study

The main aim of this study is to investigate the relationship between age of migration and common mental disorders (CMDs) diagnosed in mental health services during early adulthood (25–39 years). We include non-migrants as a comparison group to determine the difference in odds of CMDs between non-migrants and migrants who arrived in Norway during different life phases (early childhood, late childhood, adolescence, emerging adulthood and early adulthood) and who have different lengths of stay. Descendants are similar to migrants arriving during early childhood in particular, in that they grow up in a bicultural or multicultural context, are educated in the dominant country, can experience discrimination from a young age and more often grow up in low income families [23, 26, 27]. We therefore include descendants of migrants as a comparison group. Further, we also aim to investigate whether the relationship between age of migration and CMDs varies by migrant group and whether the relationship within different migrant groups is different for men and women.

## Method

### Data sources

Demographic information from the Central Population Registry and Statistics Norway was linked at an individual level, through a non-identifiable version of a personal identification number, to diagnosis information from the Norwegian Patient Registry (NPR) and The National Database for the Reimbursement of Health Expenses (KUHR). All Norwegian-born individuals and registered residents with at least six months of residence are assigned this personal number. NPR contains diagnosis information on all individuals who have had treatment in specialist services, while KUHR contains information on all patient contacts in primary care, as well as specialist practitioners who are contracted by the municipality. Only primary care contacts were extracted from KUHR for the purposes of this study. Data was linked for the years 2008–2019.

Ethical approval for this study was granted by the Regional Committee for Medical and Health Research Ethics, Southeast Norway (REK 2019/321) and all registry owners approved the use of their data. Consent to participate was not required since this study uses already existing administrative data.

### Study population

We used a dynamic study design, including all migrants (foreign born with two foreign-born parents), descendants (Norwegian born with two foreign-born parents), and non-migrants (Norwegian born with two Norwegian-born parents) who were aged 25–39 years and living in Norway at some point between 2008 and 2019. Individuals were followed from the year they turned 25 or the year they migrated to Norway (whichever came first) until the first CMD diagnosis during the study period or were censored upon death, emigration or turning 40 years old (whichever came first). The final study population included 1,936,039 individuals and 10,404,580 observations.

### Variables

*Outcome*: Common mental health disorder (CMD) (yes/no). Diagnosis information was extracted from KUHR for primary care and NPR for secondary care on a yearly basis. Individuals were coded as having a CMD if they had been diagnosed with anxiety or depression either in primary care (ICPC-2 codes: P01, P03, P74, P76, P81) or in secondary care (ICD-10 codes: F32, F33, F40, F41, F42). All other individuals were coded as not having a CMD.

### Exposures

#### Migrant status: migrants, descendants, and non-migrants

Age of migration/length of stay (time-varying): We grouped age of migration to represent different developmental periods as follows: 0–6 years (early childhood), 7–12 years (late childhood), 13–17 years (adolescence), 18–24 years (emerging adulthood) and 25 + years (early adulthood). Since all those moving in early childhood had a minimum of 19 years length of stay (25 (the youngest age in the dataset) − 6 (the maximum age of migration) = 19), we divided the other age of migration groups as having less than 19 years or 19 years or more years in Norway. Those moving in early adulthood, however, could only have a maximum of 14 years in Norway (39 (the oldest age in the dataset) − 25 (the youngest age of migration) = 14). Thus, this group was not divided by length of stay.

We also combined migrant status and age of migration/length of stay in part one of the analyses to allow comparisons between non-migrants, descendants and migrants with different ages at migration and lengths of stay: Non-migrant, descendant, early childhood migrant, late childhood migrant (< 19 years), late childhood migrant ((≥ 19 years), adolescence migrant (< 19 years), adolescence migrant (≥ 19 years), emerging adulthood migrant ((< 19 years), emerging adulthood migrant (≥ 19 years), early adulthood migrant.

#### Sex: man / woman

Migrant group: We grouped migrants based on their reason for migration and country of origin. Individuals who had come to Norway for protection or family members of individuals who had come to Norway for protection were grouped together as “refugees”. We divided the remaining migrants without refugee background into two groups: those from EEA countries, USA, Canada, Australia and New Zealand (shortened to EEA + migrants) and those from outside of the EEA, USA, Canada, Australia and New Zealand (non-EEA + migrants). See Additional file 1 for a classification of the main countries in each category.

### Covariates

Marital status (time varying): Married, never married, previously married/widowed.

Education level (time-varying): <= compulsory education, upper secondary, lower college/university education, upper college/university education.

Low-income (time varying): (Yes/No) This was based on total personal income of individuals in the dataset (from employment/business income and taxable and tax-free benefits or other transfers). In accordance with the European Union’s (EU) definition for low income, we defined low income as lower than 60% of the median income per year [41].

### Missing data

Education level: educational attainment was missing for 5.9% observations. Around 16% of individuals with at least one missing observation had a recorded education later in the dataset. We replaced missing education level in previous years with the education level in the next available year. This left 8.1% of individuals consistently missing educational attainment, 92.8% were migrants. Almost 25% of migrants had missing educational attainment. Rather than attempting imputation on, or excluding, such a large percentage, we coded them as having an unknown education level.

Low income: Income was missing for around 2.9% of observations. Around 44% occurred in the last year a person was in the study. We replaced missing values with values from the previous year in the study. Other individuals were missing the first year(s) in the study but not later years. We imputed these values with values from the first available year. This left 1.8% of individuals consistently missing income. We coded them as not having a low income, the most common value.

Migrant group: Around 10.5% of migrants were missing a reason for migration. Reason for migration has typically not been recorded for migrants from Nordic countries and more recently, not been a reporting requirement for EEA migrants. It has also only been recorded for migrants arriving in Norway from 1990 onwards. Migrants arriving before 1990 (3.1%) do, therefore, not have an official reason for migration. Most of these migrants were children or adolescents at time of migration. To retain a sizeable number of migrants arriving during childhood, we imputed reason for migration based on country of origin. We only did this if we were confident that most individuals from a particular country were likely or unlikely to have needed protection. See Additional file 2 for a detailed description of migration countries (and time points) that we imputed. This resulted in an additional 8.1% of migrants being assigned a migrant group.

### Statistical analyses

We divided and described the study population by migrant category and age of migration in the descriptive analyses. We conducted chi-square analyses/one-way ANOVAs to determine if there were significant differences across the different groups on each variable. In part one of the analyses, we conducted discrete time logistic regression analyses to investigate the relationship between age of migration and CMDs, comparing with non-migrants and descendants. We adjusted for age and age squared first, followed by sex and marital status in the second model, and education level and income in the final model.

In part two of the analyses, we only included migrants. Early adulthood migrants and refugees were set as the reference categories for age of migration/length of stay and migrant group respectively. We ran discrete time logistic regression analyses, first while adjusting for all covariates, then with an interaction term between age of migration/length of stay and migrant group. Finally, we ran a fully adjusted, full-factorial model with a three-way interaction between age of migration/length of stay, sex and migrant group. To visualise and describe the significant interactions, we ran predictive marginal analyses for all levels of the interactions of interest with other variables set to their means and plotted the predictive marginal probabilities.

We also conducted robustness analyses. First, we excluded individuals who were always missing education level. Then, for analyses with migrants only (part two), we excluded migrants whose reason for migration had been imputed.

## Results

Around 30% of the study population was made up of migrants. Only 1.3% were descendants. Table 1 shows the characteristics of the study population for the last year of study inclusion by migrant status and age of migration. Overall, only around 13% of migrants had a CMD diagnosis compared with 24% of non-migrants and 25% of descendants. Having a CMD was inversely related to age of migration, with 27% of migrants arriving during early and late childhood having had a CMD diagnosis compared with only 9% of migrants arriving during early adulthood.

**Table 1:** Characteristics of sample by migrant status and age of migration

	Total	Non-migrants	Descendants	Migrants	Late childhood	Late childhood	Adolescence	Emerging adulthood	Early adulthood
Observations	10403937	7385305	130544	2888088	107865	117025	131433	755323	1776442
*N*	1935907	1326320	25561	584026	18925	21466	25080	140637	377918
Mean years in dataset (sd)	5.4 (3.5)	5.6 (3.6)	5.1 (3.5)	4.9 (3.2)	5.7 (3.6)	5.5 (3.7)	5.2 (3.5)	5.4 (3.5)	4.7 (3)
Mean age (sd)	33.8 (5.0)	33.9 (5.2)	30.2 (4.6)	33.7(4.7)	31.52(4.9)	31.37(5.0)	31.46 (5.1)	31.15 (4.9)	35.11 (4.0)
Gender	N(%)	N(%)	N(%)	N(%)	N(%)	N(%)	N(%)	N(%)	N(%)
Men	1000893 (51.7)	680333 (51.3)	13157 (51.5)	307403 (52.6)	9805 (51.8)	11222 (52.3)	14358 (57.3)	62951 (44.8)	209067 (55.3)
Women	935014 (48.3)	645987 (48.7)	12404 (48.5)	276623 (47.4)	9120 (48.2)	10244 (47.7)	10722 (42.7)	77686 (55.2)	168851 (44.7)
Marital status^1^									
Married	700872 (36.2)	408606 (30.8)	8311 (32.5)	283955 (48.6)	5624 (29.7)	6583 (30.7)	8090 (32.3)	58455 (41.5)	205203 (54.3)
Never married	1134453 (58.6)	855421 (64.5)	16262 (63.6)	262770 (45.0)	12380 (65.4)	13634 (63.5)	15340 (61.1)	71130 (50.6)	150286 (39.8)
Previously married/widowed	100582 (5.2)	62293 (4.7)	988 (3.9)	37301 (6.4)	921 (4.9)	1249 (5.8)	1650 (6.6)	11052 (7.9)	22429 (5.9)
Education level^1^									
<=Compulsory education	344515 (17.8)	217251 (16.4)	6116 (23.9)	121148 (20.7)	5054 (26.7)	7227 (33.7)	10431 (41.6)	35613 (25.3)	62823 (16.6)
Upper secondary	614579 (31.8)	490305 (37.0)	6539 (25.6)	117735 (20.2)	4821 (25.5)	6507 (30.3)	7869 (31.4)	32931 (23.4)	65607 (17.4)
Lower college/university	548434 (28.3)	432208 (32.6)	7628 (29.8)	108698 (18.6)	4855 (25.7)	4954 (23.1)	4218 (16.8)	27587 (19.6)	67084 (17.8)
Upper college/university	271129 (14.0)	176025 (13.3)	4446 (17.4)	90658 (15.5)	2439 (12.9)	2103 (9.8)	1584 (6.3)	15682 (11.2)	68850 (18.2)
Missing	157150 (8.1)	10531 (0.8)	832 (3.3)	145787 (25.0)	1756 (2.3)	675 (3.1)	978 (3.9)	28824 (20.5)	113554 (30.0)
Low income^1^									
No	1504706 (77.7)	1131810 (85.3)	18077 (70.7)	354819 (60.8)	14144 (74.7)	15390 (71.7)	17501 (69.8)	88920 (63.2)	218864 (57.9)
Yes	431201 (20.4)	194510 (14.7)	7484 (29.3)	229207 (39.2)	4781 (25.3)	6076 (28.3)	7579 (30.2)	51717 (36.8)	159054 (42.1)
Common mental disorder	394494 (20.4)	313904 (23.7)	6268 (24.5)	74322 (12.7)	5177 (27.4)	5758 (26.8)	5680 (22.7)	22273 (15.8)	35434 (9.4)
Length of stay^1^									
< 19 years				535479 (91.7)	0 (0.0)	7624 (35.5)	16436 (65.5)	133501 (94.9)	377918 (100.0)
≥ 19 years				48547 (8.3)	18925 (100.0)	13842 (64.5)	8644 (34.5)	7136 (5.1)	0 (0.0)
Migrant group (n = 569994)									
EEA+				290510 (51.0)	4105 (29.6)	3160 (16.8)	3898 (16.5)	70758 (50.9)	208589 (55.7)
non-EEA+				151831 (26.6)	1445 (10.4)	3215 (17.0)	4450 (18.8)	35120 (25.3)	107601 (28.7)
Refugee background				127653 (22.4)	8322 (60.0)	12484 (66.2)	15289 (64.7)	33131 (23.8)	58427 (15.6)

^1^ Time-varying variables show characteristics for the last year of study inclusion

### Age of migration/length of stay and CMDs

Table 2 shows the results of the discrete time logistic regression analyses. In the unadjusted model (model 1), descendants and migrants arriving as children regardless of length of stay had significantly higher yearly odds of a CMD than non-migrants. Adolescent migrants with ≥ 19 years in Norway also had higher odds, while those with < 19 years had lower odds than non-migrants. Migrants who moved both during emerging or early adulthood and had been in Norway < 19 years had far lower odds of a CMD than non-migrants. Emerging adulthood migrants with ≥ 19 years in Norway had similar yearly odds. The yearly odds of a CMD were highest for late childhood migrants and lowest among early adulthood migrants. Within each age of migration category, migrants with longer stays had higher odds of a CMD than those with shorter stays. A similar pattern was seen after adjusting for sex and marital status (model 2). In the fully adjusted model (model 3), descendants and early childhood migrants had slightly, but still significantly, higher yearly odds of a CMD than their non-migrant counterparts (8% and 11% respectively). Migrants arriving during late childhood had around 12% higher yearly odds of a CMD if they had ≥ 19 years in Norway, while those with < 19 years had around 10% lower odds. Adolescent migrants with < 19 years in Norway had lower odds while those with ≥ 19 years in Norway had similar odds to non-migrants. Migrants moving during both stages of adulthood had significantly lower odds of a CMD than non-migrants, regardless of length of stay.

**Table 2:** Odd ratios (OR) and 95% confidence intervals (CI) for common mental disorder

	Model 1	Model 2	Model 3	
	OR (95% CI)	OR (95%)	OR (95% CI)	
Non-migrant	1	1	1	
Descendant	1.14 (1.10–1.16)***	1.16 (1.12–1.21)***	1.08 (1.04–1.12)***	
Early chilhood	1.22 (1.17–1.28)***	1.22 (1.17–1.28)***	1.11 (1.06–1.16)***	
Late childhood, < 19 years	1.27 (1.06–1.20)***	1.13 (1.06–1.20)**	0.90 (0.85–0.96)***	
Late childhood, ≥ 19 years	1.37 (1.30–1.45)***	1.39 (1.31–1.46)***	1.12 (1.06–1.18)***	
Adolescence, < 19 years	0.92 (0.87–0.96)***	0.97 (0.92–1.01)	0.72 (0.69–0.75)***	
Adolescence, ≥ 19 years	1.19 (1.10–1.28)***	1.24 (1.14–1.34)***	0.94 (0.87–1.01)	
Emerging adulthood, < 19 years	0.50 (0.49–5.11)***	0.48 (0.47–0.49)***	0.45 (0.45–0.47)***	
Emerging adulthood, ≥ 19 years	1.10 (0.99–1.23)	0.99 (0.89–1.10)	0.81 (0.73–0.90)***	
Early adulthood	0.29 (0.25–0.26)***	0.29 (0.29–0.30)***	0.35 (0.34–0.35)***	
Woman		2.45 (2.42–2.48)***	2.56 (2.54–2.59)***	
Marital status				
Married		1	1	
Never married		1.31 (1.30–1.32)***	1.28 (1.27–1.29)***	
Previously married/widowed		2.71 (2.66–2.77)***	2.28 (2.24–2.33)***	
Education level				
<=Compulsory education			4.10 (4.03–4.18)***	
Upper secondary			1.99 (1.95–2.02)***	
Lower college/university			1.38 (1.35–1.40)***	
Upper college/university			1	
Missing			0.82 (0.79–0.85)***	
Low income			1.27 (1.26–1.29)***	
Observations per model = 10,403,937				

*N* = 1,935,907

**p < 0.01; ***p < 0.001

### Age of migration and CMDs by migrant group and sex

By the last year of study inclusion, CMDs were more common among refugees (19.8%) than EEA (9.8%) and non-EEA migrants (11.3%). In a discrete-time logistic regression analyses with EEA migrants at baseline, the differences between EEA and non-EEA migrants were still significant after adjusting for sex, marital status, education level and low income (OR = 1.14, 95% CI: (1.11–1.17)), as were the differences between EEA and refugee migrants (OR = 2.02, 95% CI: (1.97–2.07)). Thus, our two non-refugee groups were not combined in analyses with migrants only.

**Fig. 1:**
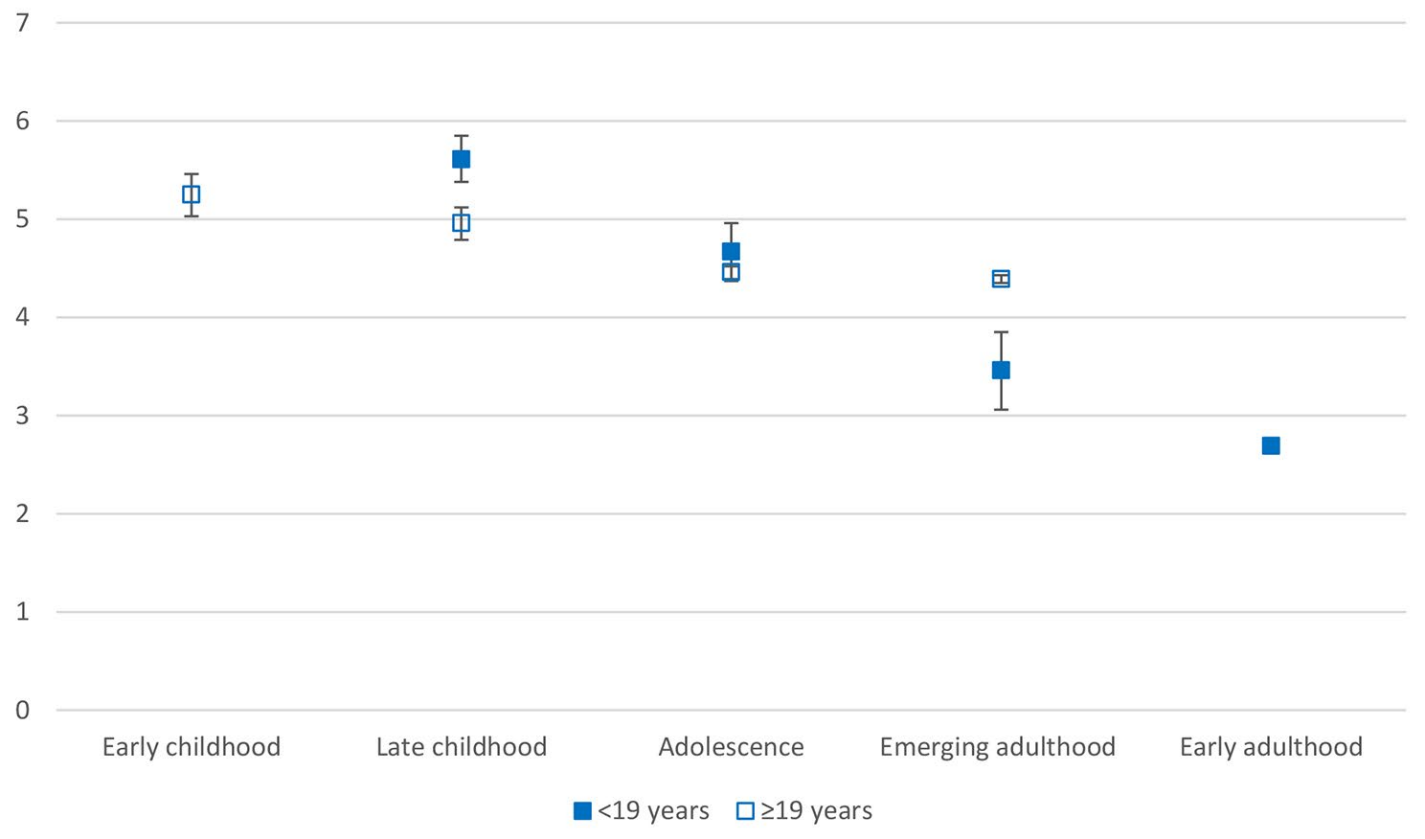
Average marginal predicted probabilities for CMDs by age of migration and length of stay

Table 3, model 1 shows the main effects of age of migration/length of stay, migrant group and sex, after adjusting for marital status, education level and low income. Compared with those migrating during early adulthood, all migrants arriving during an earlier stage had higher yearly odds of CMDs. Odds were highest for those moving during late childhood with less than 19 years in Norway. The odds generally decreased with increasing age of migration. Women had more than double the yearly odds of a CMD compared with men. Both groups of non-refugees had lower yearly odds of a CMD than refugees. We plotted average marginal predicted probabilities for CMDs by age of migration (Fig. 1). To visualise whether the relationship between age of migration and CMDs was the same regardless of length of stay, we plotted those with < 19 years in Norway separately from those with ≥ 19 years in Norway. The figure shows that the there is a clear negative relationship between age of migration and CMDs among migrants with < 19 years in Norway. For those with ≥ 19 years in Norway, the relationship was weak, though the probability of CMDs was still slightly higher among those moving in childhood than in adolescence or emerging adulthood. Notably, the probability of CMDs was higher among those with ≥ 19 years in Norway than those with < 19 years for those arriving during emerging adulthood, but lower for migrants arriving during late childhood. There was no difference in predicted probabilities of CMDs by length of stay for the adolescent group.

**Table 3:** Fully adjusted odds ratios (OR) and 95% confidence intervals (CI) with interactions for CMDs 1;2

	Model 1 OR (95% CI)	Model 2 OR (95% CI)	Model 3 OR (95% CI)	Model 4 Or (95% CI)
Age of migration				
Early childhood	2.22 (2.11–2.34)***	1.89 (1.77–2.02)***	2.18 (2.02–2.35)***	1.49 (1.35–1.64)***
Late childhood (< 19 years)	2.42 (2.29–2.55)***	1.92 (1.79–2.06)***	2.48 (2.29–2.70)***	1.65 (1.49–1.82)***
Late childhood (≥ 19 years)	2.08 (1.96–2.20)***	1.67 (1.55–1.79)***	2.13 (1.97–2.32)***	1.39 (1.26–1.53)***
Adolescence (< 19 years)	1.92 (1.84–2.01)***	1.58 (1.50–1.68)***	2.04 (1.91–2.17)***	1.39 (1.29–1.50)***
Adolescence (≥ 19 years)	1.82 (1.68–1.97)***	1.50 (1.36–1.64)***	2.07 (1.86–2.30)***	1.37 (1.21-0.55)***
Emerging adulthood (< 19 years)	1.34 (1.31–1.37)***	1.10 (1.05–1.15)***	1.39 (1.34–1.44)***	1.12 (1.05–1.19)***
Emerging adulthood (≥ 19 years)	1.78 (1.60–1.99)***	1.37 (1.17–1.60)***	2.15 (1.79–2.58)***	1.44 (1.15–1.80)***
Early adulthood	1	1	1	1
Migrant group				
Refugees	1	1	1	1
EEA+	0.61 (0.59–0.63)***	0.52 (0.50–0.54)***	0.61 (0.50–0.62)***	0,36 (0,34 − 3,74)***
non-EEA+	0.65 (0.63–0.67)***	0.54 (0.52–0.56)***	0.65 (0.63–0.67)***	0,52 (0,49 − 0,55)***
Women	2.10 (2.06–2.15)***	2.10 (2.05–2.14)***	2.16 (2.10–2.22)***	1.39 (1.32–1.47)***
Migrant group*age of migration				
EEA+*early childhood		1,14 (1,01–1,28)*		1.64 (1.37–1.95)***
non-EEA+*early childhood		1,85 (1,57 − 2,18)***		1.98 (1.55–2.54)***
EEA+*late childhood, < 19 years		1,68 (1,44 − 1,96)***		2.31 (1.82–2.93)***
non-EEA+*late childhood, < 19 years		1.61 (1.40–1.85)***		1.45 (1.16–1.81)**
EEA+*late childhood, ≥ 19 years		1,76 (1,51 − 2,05)***		2.53 (2.02–3.18)***
non-EEA+* late childhood, ≥ 19 years		1.45 (1.32–1.59)***		1.47 (1.15–1.88)***
EEA+*adolescence, < 19 years		1,54 (1,36 − 1,75)***		1.94 (1.62–2.36)***
non-EEA+*adolescence, < 19 years		1,43 (1,28 − 1,59)***		1.39 (1.18–1.64)***
EEA+*adolescence, ≥ 19 years		1,79 (1,40 − 2,30)***		2.66 (1.85–3.82)***
non-EEA+*adolescence, ≥ 19 years		1,38 (1,11 − 1,71)**		1.38 (1.01–1.88)***
EEA+*emerging adulthood, < 19 years		1.28 (1.21–1.35)***		1.23 (1.13–1.33)***
non-EEA+*emerging adulthood, < 19 years		1.36 (1.28–1.44)***		1.41 (1.27–1.55)***
EEA+*emerging adulthood, ≥ 19 years		1.34 (1.01–1.77)*		1.57 (0.91–2.70)***
non-EEA+*emerging adulthood, ≥ 19 years		1.79 (1.39–2.31)***		1.91 (1.19–3.07)***
Sex*age of migration				
women*early childhood			1.04 (0.94–1.15)	1.60 (1.41–1.82)***
women*late childhood, < 19 years			0.95 (0.86–1.06)	1.37 (1.20–1.57)***
women*late childhood, ≥ 19 years			0.95 (0.85–1.06)	1.41 (1.23–1.62)***
women*adolescence, < 19 years			0.89 (0.82–0.97)**	1.26 (1.12–1.40)***
women*adolescence, ≥ 19 years			0.77 (0.68–0.89)**	1.12 (0.93–1.35)
women*emerging adulthood, < 19 years			0.94 (0.90–0.98)*	1.01 (0.92–1.09)
women*emerging adulthood, ≥ 19 years			0.75 (0.60–0.94)*	0.92 (0.67–1.24)
Sex*migrant group				
women*EEA+				2.17 (2.03–2.31)***
women*non-EEA+				1.19 (1.10–1.28)***
Migrant group*age of migration*sex				
EEA+*early childhood*woman				0.47 (0.37–0.60)***
non-EEA+*early childhood*woman				0.80 (0.57–1.11)
EEA+*late childhood < 19 years*woman				0.52 (0.38–0.71)***
non-EEA+*late childhood < 19 years*woman				1.07 (0.81–1.42)
EEA+* late childhood, ≥ 19 years *woman				0.49 (0.36–0.66)***
non-EEA+* late childhood, ≥ 19 years *woman				0.94 (0.68–1.01)
EEA+*adolescence, < 19 years*woman				0.63 (0.49–0.80)***
non-EEA+*adolescence, < 19 years*woman				0.98 (0.79–1.23)
EEA+*adolescence, ≥ 19 years*woman				0.49 (0.30–0.81)***
non-EEA+*adolescence, ≥ 19 years*woman				0.96 (0.62–1.48)
EEA+*emerging adulthood, < 19 years*woman				0.96 (0.86–1.07)
non-EEA+*emerging adulthood, < 19 years*woman				0.94 (0.83–1.06)
EEA+*emerging adulthood, ≥ 19 years*woman				0.74 (0.39–1.40)
non-EEA+*emerging adulthood, ≥ 19 years*woman				0.94 (0.53–1.66)
Observations per model = 2,814,355				

*N* = 569,994

^1^ Common mental disorder; ^2^All models adjusted for marital status, education level and low income; **p < 0.01; ***P > 0.001

**Fig. 2:**
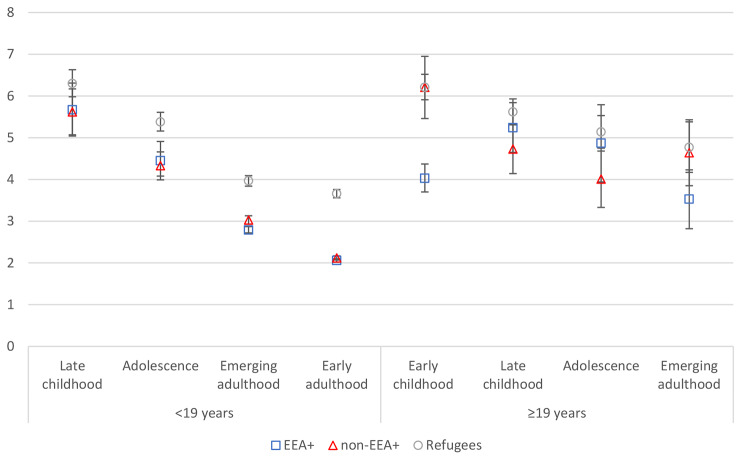
Average marginal predicted probabilities for CMDs by age of migration/length of stay and migrant group

In model 2, we investigated the interaction between age of migration/length of stay and migrant group. The addition of the interaction terms improved the fit of the model (χ^2^(14) = 265.82, *p* < 0.001). All interaction terms were significant. The higher odds ratio for each indicates that the relationship between age of migration/length of stay and CMDs is stronger for both the non-refugee groups compared with refugees. We plotted average marginal predicted probabilities (expressed as percentage) for all levels of the interaction (Fig. 2), again with separate plots for illustrative purposes for those with < 19 and ≥ 19 years in Norway. For migrants with ≥ 19 years in Norway, there was no clear association between age of migration and CMDs, regardless of migrant group. For those with < 19 years in Norway, there was a negative association between age of migration and CMDs. The relationship appeared, however, to peter out for refugees, with little difference between those arriving during emerging or early adulthood. The higher probability of CMDs for refugees compared with the two other groups was not apparent during late childhood but appeared with increasing age of migration. However, the probably of a CMD within each age of migration category did not differ considerably by length of stay, with the exception of those those arriving during emerging adulthood, where longer stays were associated with higher probability. The figure also shows that except for in early childhood, there was no significant difference in average predicted probability of a CMD for EEA + and non-EEA + migrants.

**Fig. 3:**
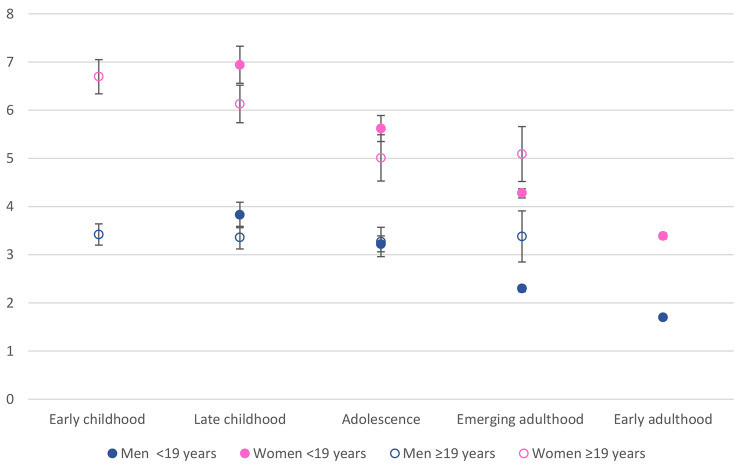
Average marginal predicted probabilities for CMDs by age of migration/length of stay and sex

**Fig. 4:**
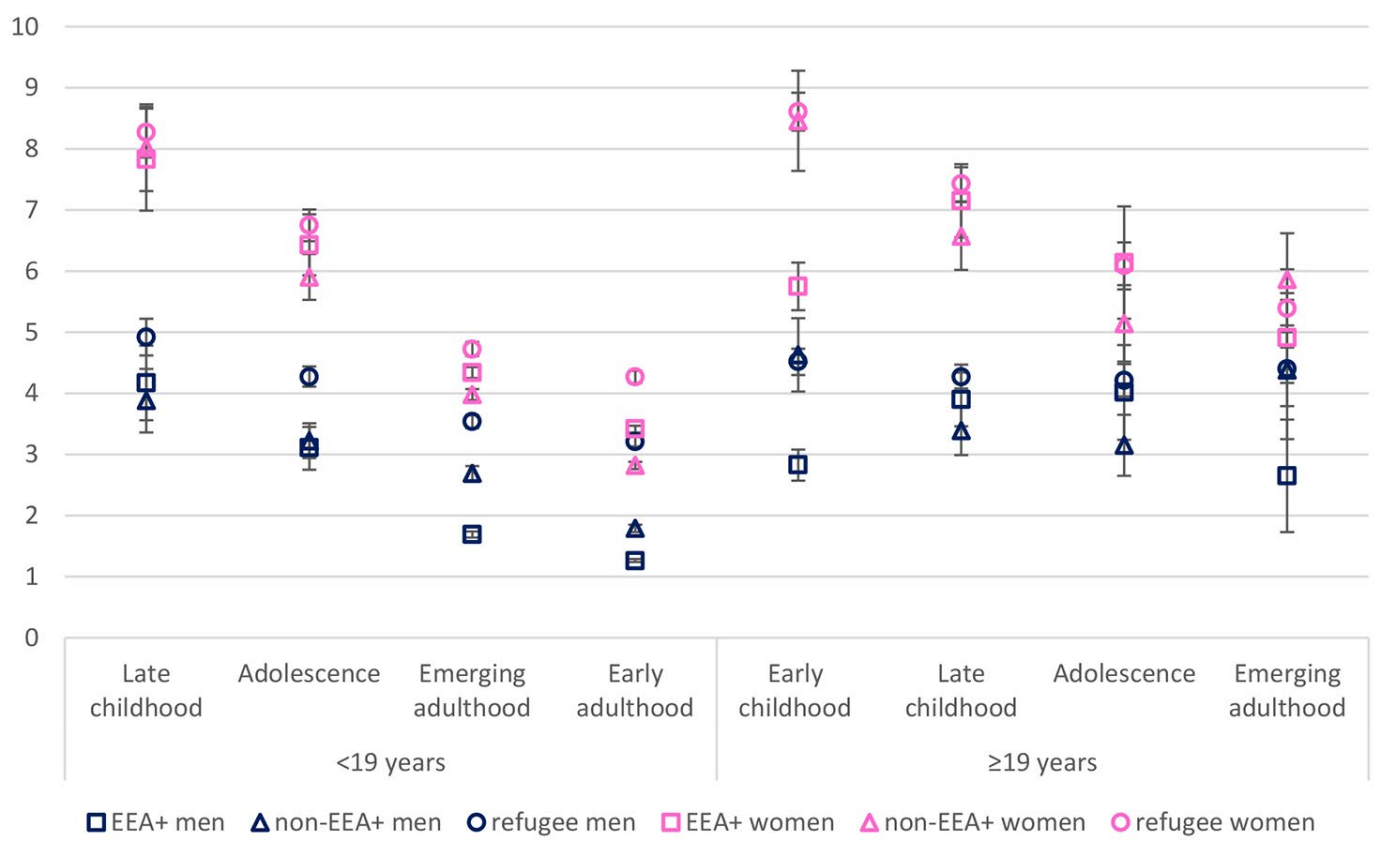
Average marginal predicted probabilities for CMDs: age of migration/length of stay, migrant group and sex

In model 3, we included an interaction term between age of migration and sex. Compared with the fully adjusted model with no interaction, the addition of the interaction improved the fit of the model (χ^2^(7) = 26,1, *p* < 0,001). There were significant interactions for adolescence and emerging adulthood, suggesting that the differences in odds of a CMD between adolescence and emerging adulthood compared with early adulthood were greater for women than for men. Plotting average marginal predicted probabilities confirmed this (Fig. 3). Additionally, the figure shows that age of migration has a negative relationship with CMDs for women with ≥ 19 years in Norway, while there is no association for men. For migrants with < 19 years in Norway, there is a negative association for both men and women, but it appears slightly stronger for women.

Finally, in model 4, we investigated whether the interaction between age of migration and sex was dependent on migrant group. Compared with model 2, the addition of the interaction term again improved the fit of the model (χ2(x) = 1025.69, *p* < 0.001). There were five significant interaction terms in the three-way interaction, all pertaining to EEA + migrants. The odds ratios indicate that the sex difference in yearly odds between early and late childhood and adolescent migrants differs for EEA + migrants compared with refugees. This appeared to be the case for both those with < 19 years and ≥ 19 years in Norway. To visualise this three-way interaction, together with the two-way interactions, we calculated and plotted adjusted average predicted marginal probabilities (expressed as percentage) for CMDs for the different migrant groups and ages of migration/length of stay for both men and women (Fig. 4). Among refugees and non-EEA + migrants, the sex difference in CMDs appears larger among those migrating as children and adolescents than those migrating as adults. This was regardless of length of stay. Thus, the relationship between age of migration and CMDs appears stronger for non-EEA + migrant women and refugee women compared with men. For EEA + migrants, however, the pattern is similar for both men and women, with only a slight narrowing of the sex gap in CMDs from childhood through to adulthood. When looking at differences across groups within each sex, the differences in predicted probability of CMDs among those with < 19 years in Norway becomes larger for refugee men compared with EEA + men with increasing age of migration. Women, in contrast, differ only among those arriving during early adulthood. Among those with ≥19 years in Norway, the differences across groups are most apparent among those arriving during early childhood, with both EEA + men and women having lower probability of a CMD compared to the other migrant groups.

### Robustness analyses

When we excluded individuals with missing education level (Additional file 3, Tables 1 and 2) descendants, early childhood and late childhood migrants with ≥ 19 years in Norway had higher yearly odds of a CMD compared with non-migrants as in the main analyses. All other groups had lower or the same yearly odds. This differed slightly from the main analyses where those moving in late childhood with < 19 years and adolescent migrants with ≥ 19 years also had higher odds. In analyses with migrants only, all the same interaction terms were still significant, with only two exceptions.

When we excluded migrants with an imputed migrant group (8.1%), the findings were similar to the main analyses (Additional file 4). The yearly odds of CMDs decreased with increasing age. In model 1, those who moved during emerging adulthood who had been in Norway ≥ 19 years had lower odds, in contrast to in the main analysis where they had higher odds. In model 2, the interaction terms were still significant, indicating a weaker relationship between age of migration and CMDs for refugees compared with the other two groups. However, the difference in yearly odds of a CMD between those migrating during early childhood and early adulthood appeared strongest for EEA + migrants (EEA+*childhood), while in the main analyses it was strongest for non-EEA + migrants compared with refugees. In model 3, the interaction term women*adolescence < 19 years was only borderline significant. Finally, in model 4, the same interaction terms were significant as in the main analyses, though again, the effect size of childhood and adolescence for EEA + migrants appeared stronger. This may be due to the large percentage of EEA + migrants (mainly from Nordic countries) being excluded here compared with in the main analysis. The three-way interaction was consistent with the findings in the main analyses.

Overall, the robustness analyses generally confirm our confidence in the findings of the imputed data.

## Discussion

In this study, we investigated differences in yearly odds of diagnosed CMDs during early adulthood based on age of migration. Our findings show a general negative association between age of migration and length of stay, but that the effect appears to be weaker among migrants with long stays. This differs from findings on psychotic disorders, where the negative relationship with age of migration does not appear to attenuate with longer duration [42, 43]. It is possible that increased exposure to socioeconomic inequalities, discrimination and other long-term migratory related stressors increases the risk of CMDs over time, also for migrants arriving during adulthood. Indeed, we found that the probability of a CMD is greater for migrants arriving during emerging adulthood with 19 or more years in Norway than for those with less than 19 years in Norway (Fig. 1).

Importantly, not only did migrants arriving during early childhood or late childhood with more than 19 years in Norway have slightly higher odds of CMDs than non-migrants but descendants did too. Thus, it may not be the experience of migration itself which increases the risk of CMDs in early adulthood for childhood migrants relative to non-migrants, but rather the experience of growing up with a migrant background and the challenges it entails. Multiple socio-economic disadvantages, discrimination and acculturative stress experienced during a sensitive period and cumulating over time can contribute to poorer future mental health [23, 27–29].

Our findings are in contrast to the Swedish study which found that migrants arriving during childhood reported better mental health than migrants arriving during adulthood [5], though similarly, the effect decreased with increasing length of stay. The Swedish study was collected at one point in time and based on self-report data, which may be subject to both recall and selection bias. In the current register study, we were able to follow individuals for up to 12 years and had national coverage, meaning there was no selection or recall bias. Honkaniemi and colleagues focused on psychological distress, while our measure of CMDs was defined as a diagnosis set in healthcare services. As such, to be identified as having a CMD, migrants need to have used primary or secondary healthcare services. Migrants can experience major barriers to care including language barriers, stigma, feelings of mistrust for healthcare providers and difficulties in navigating the system [44–48]. Thus, we are unlikely to have detected all CMDs in our study.

Barriers, and lower levels of accessing care, could therefore also explain the relationship between age of migration and diagnosed CMDs. Both migrants arriving during childhood and descendants are likely to be fluent in the receiving country’s language and have experience in using the healthcare system. Language proficiency is associated with help-seeking [49]. Further, some childhood migrants and descendants, depending on migrant background, may also have grown up in a context (through school and social networks) with more openness around mental disorder than what migrants arriving as adults have experienced. This may increase help-seeking [45]. Theoretically then, the barriers for accessing mental health care should be lower for migrants arriving as children (and descendants) than for migrants arriving as adults. This may particularly be the case for migrants from countries where stigma surrounding mental disorder is high. Thus, our finding that the odds of diagnosed CMDs decrease with increasing age of migration may reflect childhood migrants’ greater propensity to seek help when experiencing CMD symptoms, rather than their actual mental health status. In other words, the under identification of CMDs may be greater among migrants arriving as adults than as children. Further, these barriers are likely to be greatest among migrants with shorter stays and, for many, they will reduce overtime as familiarity with the health system and language proficiency increases. Thus, improved access to care, rather than a decline in mental health, over time, could be an alternative explanation for why migrants arriving during emerging adulthood with 19 or more years in Norway had a higher probability of CMDs than those with less than 19 years.

We also investigated whether the relationship between age of migration and CMDs differed for across migrant groups. For migrants with less than 19 years in Norway, the negative association between age of migration and CMDs appeared slightly weaker among refugees, since the difference in probability between refugees and other groups increased with increasing age of migration. This could be due to an overall weaker healthy migrant effect for refugees due to the lower level of voluntary migration as well as the increased risk of traumatic experiences. Moreover, we also saw this weaker association between age of migration and CMDs among refugees was particularly the case for men. Men may often report higher levels of traumatic experiences [50] and are more likely to arrive as asylum-seekers, while women may more often follow though family reunification. Thus, refugee men may be less prepared for migration and more often face an uncertain future. Experiencing a prolonged asylum process is also associated with increased risk of CMDs [51]. With the exception of unaccompanied minors, it is possible that adults, particularly men, experience more stress related to the asylum process than any children travelling with them, which would further heighten the risk of CMDs among those arriving as adults.

Among migrants living in Norway for 19 years or more, however, the probability of a CMD diagnosis was similar for all three groups at different ages of migration, with the exception of EEA + migrants arriving during early childhood. This group had a lower probability compared with the other two groups. This lower level of CMDs among EEA + migrants arriving during early childhood applied to both men and women in the three-way interaction analyses (Fig. 4). Previous research suggests that migrants arriving at a younger age tend fare better in terms of educational attainment and labour market participation [13], and that these factors are protective of CMDs [14, 15]. Yet, childhood migrants can experience more cultural conflict and discrimination which can instead increase the risk of CMDs. Given the closer cultural ties to Norway in many EEA + countries, it is possible that this group of childhood migrants experience lower levels of cultural conflict as well as lower levels of discrimination due to being a less visible group of migrants. Thus, future research should investigate if the mental health benefits of any socioeconomic advantage that childhood migrants may experience is mitigated by acculturative stress or experiences of discrimination.

Among those with less than 19 years in Norway, there was little difference in the relationship between age of migration and CMDs for men and women from EEA + countries. In contrast, the difference in predicted probability of CMDs appeared to narrow with increasing age of migration for refugees and non-EEA + migrants. One explanation for this is that girls with parents from some countries outside of the EU, USA, Canada, Australia and New Zealand, may experience less freedom than boys, greater levels of acculturative stress due to conflicting gender norms and more negative social control [37–39]. This may have an impact on young migrant women’s mental health during early adulthood. Yet, a closer look across the different migrant groups suggests that refugee and non-EEA + women migrating as girls did not have an increased probability of CMDs relative to their EEA + counterparts (except for in early childhood as previously discussed). There was only a significant difference in probability for those migrating as adults. Further, the difference in probability across the migrant groups appears to increase with increasing age of migration for men more than for women, and particularly refugee men. Men in these two groups may often come from countries where men are traditionally the breadwinners. It is possible that they therefore feel more pressure to provide for their families but at the same time can often experience obstacles such as unrecognised qualifications, underemployment, language barriers and discrimination on the job market [52, 53]. Thus, difficulties in obtaining employment and resulting financial worries could have a greater impact on refugee and non-EEA + men arriving as adults from than for women. This could result in a narrowing of the gender gap in these groups with increasing age of migration, especially for refugee men who may also have experienced pre-migration trauma and a stressful asylum-seeking process.

There are several implications for this study. Since growing up with a migrant background may pose an extra challenge and increase the risk of CMDs during early adulthood compared with non-migrants, mental health promotion and prevention programs should include consideration to children with a migrant background. Such programs should be culturally adapted and implemented with a focus on the specific needs of the target group. Parents of migrant and descendant children could be included in such programs to help them understand the challenges their child can face when growing up with conflicting norms and roles in and outside of the home. This may be particularly important for those who originating from countries outside of the EEA, USA, Canada, Australia and New Zealand. Health care professionals should also be aware that descendants and migrants arriving from non-EEA countries at a young age may be at higher risk of CMDs than their majority and adulthood migrant counterparts. They need the competency to identify and understand the challenges that these young people face, so they can offer appropriate interventions or support in culturally meaningful ways. Our findings also confirm the need for continued focus on the mental health of refugees, who, are at increased risk of CMDs. Importantly, CMD diagnoses appeared more common among migrants with longer stays. On the one hand, this could indicate that long-term exposure to stressors such as discrimination or social inequality increase the risk of CMDs. Thus, efforts to reduce inequalities and discrimination in society could help prevent CMDs among migrants. On the other hand, barriers to using health care for migrants arriving after late childhood may also partly explain the negative relationship between age of migration and diagnosed CMDs. Thus, greater efforts to reduce barriers, with a particular focus on migrants arriving as adults, must be made. Interventions improving language skills, health literacy and understanding of the healthcare service as well as the competency of health care professionals in identifying mental health problems in different migrant groups could potentially lead to improved help-seeking, identification and treatment of CMDs among newly arrived adult migrants.

### Limitations

Data on healthcare use prior to 2008 was not available, so we were unable to see if a person was previously diagnosed with a CMD, or if the onset occurred before inclusion at 25 years. Although young adulthood is a common time for onset of CMDs, adolescence and emerging adulthood are also risk periods [54]. There may also be an under-identification of CMDs among individuals who were already aged 25 + in 2008, and for migrants who arrived in Norway after 2008. This could be particularly the case for migrants arriving both during early adulthood and after 2008. Thus, the difference in odds of CMDs between migrants arriving as children and as adults could be slightly overestimated. On the other hand, the analysis accounts for the number of years an individual contributes to the dataset, and thus those who are in the dataset for a shorter period have less impact on the results.

Although we offer several explanations for our findings, we are unable to tease out how much of the relationship between age of migration and CMDs can be attributed to each explanation (growing up with a migrant background and the associated acculturation stress, lower health selection among younger migrants, declining mental health with increasing length of stay or greater barriers for migrants arriving as adults). Future studies could have a longer follow-up time during adulthood to determine whether migrants arriving during adulthood also end up with increased risk of CMDs after many years in Norway or if this is only for those migrating at younger ages. Studies could also utilise different data sources, such as self-reported data or clinical interviews to complement our study. However, such studies are often subject to selection bias. Suicide is also often attributed to mental disorder, including depression. Assessing the relationship between age of migration and suicide could help to discern if the lower risk among those migrating as adults is due to this group being less likely to seek care. If the pattern was far weaker than in the current study, it might suggest that many migrants arriving as adults fail to seek help for mental disorders.

Finally, there are several confounders that we have been unable to account for, such as pre-migration negative life events including traumatic experiences, parental mental disorder as well as post-migration stressors such as perceived discrimination or negative social control. These may help explain the relationship between CMDs and age of migration.

## Conclusions

Migrants arriving as children with long stays and descendants of migrants have higher odds of a CMD diagnosis during early adulthood than their non-migrant counterparts. We argue that this difference is more likely to be due to the challenges of growing up between two cultures and the experience of discrimination or social exclusion than the migration experience itself. The decline in risk of CMDs with increasing age of migration could also be due to a greater health selection effect among adults compared with children. Yet, this health advantage decreases with increasing length of stays. Prevention programs fostering positive mental health and social inclusion from a young age could have universal benefits, especially for children with a migrant background from outside of EEA, USA, Canada, Australia and New Zealand. Barriers to health care should also be addressed, particularly for migrants migrating as adults.

### Electronic supplementary material

Below is the link to the electronic supplementary material.

Supplementary Material 1

Supplementary Material 2

Supplementary Material 3

Supplementary Material 4

## Data Availability

The datasets generated and analysed for the current study are not publicly available for data protection reasons. However, the data that support the findings of this study may be available from Statistics Norway and HELFO if ethical approval is granted.

## Declarations

### Disclaimer

The interpretation and reporting of the data obtained from the different registers utilised are the sole responsibility of the authors, and no endorsement by any of the registries is intended nor should be inferred.

## Abbreviations

CMD: Common mental disorder
EEA: European Economic Area
EU: European Union
OR: Odds ratio
CI: Confidence interval

## References

[1] Close C, Kouvonen A, Bosqui T, Patel K, O’Reilly D, Donnelly M. The mental health and wellbeing of first generation migrants: a systematic-narrative review of reviews. Global Health. 2016;25(1):47. 10.1186/s12992-016-0187-3 PMC4997738 27558472

[2] Gilliver SC, Sundquist J, Li X, Sundquist K. Recent research on the mental health of immigrants to Sweden: a literature review. Eur J Public Health. 2014;24(suppl1):72–9. 25108001 10.1093/eurpub/cku101

[3] Brendler-Lindqvist M, Norredam M, Hjern A. Duration of residence and psychotropic drug use in recently settled refugees in Sweden - a register-based study. Int J Equity Health. 2014;13:122. 25526935 10.1186/s12939-014-0122-2 PMC4297375

[4] Anderson KK, Edwards J. Age at migration and the risk of psychotic disorders: a systematic review and meta-analysis. Acta Psychiatrica Scandinavica. 2020;141(5):410–20. 31903545 10.1111/acps.13147

[5] Honkaniemi H, Juarez SP, Katikireddi SV, Rostila M. Psychological distress by age at migration and duration of residence in Sweden. Soc Sci Med. 2020;1:112869. 10.1016/j.socscimed.2020.112869 PMC8325349 32120203

[6] Islam F, Khanlou N, Tamim H. South Asian populations in Canada: migration and mental health. BMC Psychiatry. 2014;14(1):154. 24884792 10.1186/1471-244X-14-154 PMC4052282

[7] Nesterko Y, Braehler E, Grande G, Glaesmer H. Life satisfaction and health-related quality of life in immigrants and native-born germans: the role of immigration-related factors. Qual Life Res. 2013;22(5):1005–13. 22843126 10.1007/s11136-012-0239-y

[8] Patterson B, Kyu HH, Georgiades K. Age at Immigration to Canada and the occurrence of Mood, anxiety, and Substance Use disorders. Can J Psychiatry. 2013;58(4):210–7. 23547644 10.1177/070674371305800406

[9] Guo M, Stensland M, Li M, Dong X, Tiwari A, Mundt A, Busch MA, Nickels E, Heimann HM, Rapp MA. Gerontologist. 2019;59(5):865–76. American Psychiatric Association, Angel, JL, Angel, RJ, Angel, JL, Buckley, CJ, Sakamoto, A, Batalova, J, Benjet, C, Bromet, E, Karam, EG, Kess, editor.29931059

[10] Lam J, Yip T, Gee G. The physical and mental health effects of age of immigration, age, and perceived difference in social status among first generation Asian americans. Adler NE Epel E Castellazzo G Ickovics J Aiken L West S Alegria M Takeuchi D Canino G Duan N Shrout P Meng X Escobar J … Alegria M Vila D Woo M Canino G Takeuchi D Vera M Shrout P … Angel J Editor Special Issue: Secondary Anal Natl Latino Asian Am Study (NLAAS) Dataset-Part I. 2012;3(1):29–43.

[11] Mossakowski KN. Are immigrants healthier? The case of Depression among Filipino americans. Soc Psychol Q. 2007;70(3):290–304.

[12] Yang FJ. Is childhood migration a mental health risk? Exploring health behaviors and psychosocial resources as pathways using the cross-sectional Canadian Community Health Survey. Soc Sci Res. 2019;1:102303. 10.1016/j.ssresearch.2019.04.016 31422841

[13] Hermansen AS. Age at arrival and life chances among childhood immigrants. Demography. 2017;54:201–29. 28054254 10.1007/s13524-016-0535-1

[14] Kondirolli F, Sunder N. Mental health effects of education. Health Econ. 2022;31(Suppl 2):22. 35797349 10.1002/hec.4565 PMC9796491

[15] Modini M, Joyce S, Mykletun A, Christensen H, Bryant RA, Mitchell PB, et al. The mental health benefits of employment: results of a systematic meta-review. Australas Psychiatry. 2016;24(4):331–6. 26773063 10.1177/1039856215618523

[16] Shields-Zeeman L, Smit F. The impact of income on mental health. Lancet Public Health. 2022;7(6):e486–7. 35660205 10.1016/S2468-2667(22)00094-9

[17] Stafford AM, Sojda D, Mercado Emerson M, Nagy GA, McCabe BE, Gonzalez-Guarda RM. Age of immigration and depressive symptoms among young adult Latinx immigrants: A test of explanatory models., Abraido-Lanza AF, Dohrenwend BP, Ng Mak DS, Turner JB, Alegria M, Alvarez K, DiMarzio K, Alegria M, Canino G. Shrout, PE, Woo, M, Duan, N, Vila, D, Torres, M, Chen, C, Meng, XL, Alegria, M, Sribney, W, Woo, M, Torres, M, Guarnacc, editor. Hispanic Health Care International. 2023;21(1):14–21. 10.1177/15404153221088929 PMC10236318 35317632

[18] Sisk LM, Gee DG. Stress and adolescence: vulnerability and opportunity during a sensitive window of development. Curr Opin Psychol. 2022;44:286–92. 34818623 10.1016/j.copsyc.2021.10.005 PMC9007828

[19] Jones NL, Gilman SE, Cheng TL, Drury SS, Hill CV, Geronimus AT. Life Course approaches to the causes of Health disparities. Am J Public Health. 2019;109(Suppl 1):S48. 30699022 10.2105/AJPH.2018.304738 PMC6356123

[20] Andersen SH, Steinberg L, Belsky J. Beyond early years versus adolescence: the interactive effect of adversity in both periods on life-course development. Dev Psychol. 2021;57(11):1958–67. 34914456 10.1037/dev0001247

[21] Schwartz SJ, Hardy SA, Zamboanga BL, Meca A, Waterman AS, Picariello S, et al. Identity in young adulthood: links with mental health and risky behavior. J Appl Dev Psychol. 2015;36:39–52. 34334855 10.1016/j.appdev.2014.10.001 PMC8319849

[22] Spencer N. The social determinants of child health. Paediatrics Child Health. 2018;28(3):138–43.

[23] Oppedal B, Toppelberg CO. Acculturation developmentand the acquisition of culture competence. In: Sam DL, Berry JW, editors. The Cambridge Handbook of Acculturation Psychology [Internet]. 2nd ed. Cambridge: Cambridge University Press; 2016 [cited 2023 Dec 13]. pp. 71–92. (Cambridge Handbooks in Psychology). https://www.cambridge.org/core/books/cambridge-handbook-of-acculturation-psychology/acculturation-developmentand-the-acquisition-of-culture-competence/1F4D07B7F57BE825430A6984AEDD608A.

[24] Rogers-Sirin L, Ryce P, Sirin S, Acculturation. Acculturative Stress, and Cultural Mismatch and Their Influences on Immigrant Children and Adolescents’ Well-Being. In: Dimitrova M, Bender M, van de Vijver F, editors. Global Perspectives on Well-Being in Immigrant Families. 2014. pp. 11–30.

[25] Sabatier C, Berry J. The role of family acculturation, parental style, and perceived discrimination in the adaptation of second-generation immigrant youth in France and Canada. Eur J Dev Psychol - EUR J DEV PSYCHOL. 2008;5:159–85.

[26] Normann TM. Children who grow up in low-income households [Internet]. Oslo-Kongsvinger: Statistics Norway; 2021. (Children in low income households). https://www.ssb.no/inntekt-og-forbruk/inntekt-og-formue/artikler/barna-som-vokser-opp-i-lavinntekt.

[27] Stevens GWJM, Vollebergh WAM. Mental health in migrant children. J Child Psychol Psychiatry. 2008;49(3):276–94. 18081765 10.1111/j.1469-7610.2007.01848.x

[28] Thomas Tobin CS, Moody MD. Does early life racial discrimination explain a Mental Health Paradox among black adults? J Aging Health. 2021;33(5–6):396–408. 33530841 10.1177/0898264320988187

[29] Astell-Burt T, Maynard MJ, Lenguerrand E, Harding S. Racism, ethnic density and psychological well-being through adolescence: evidence from the determinants of adolescent Social Well-Being and Health longitudinal study. Ethn Health. 2012;17(1–2):71–87. 22332834 10.1080/13557858.2011.645153 PMC3379740

[30] Kennedy S, Kidd MP, McDonald JT, Biddle N. The healthy immigrant effect: patterns and evidence from four countries. Int Migration Integr. 2015;16(2):317–32.

[31] Fennelly K. The healthy migrant effect. Minn Med. 2007;90(3):51–3. 17432759

[32] Bhugra D. Migration and mental health. Acta Psychiatrica Scandinavica. 2004;109(4):243–58. 15008797 10.1046/j.0001-690x.2003.00246.x

[33] Elshahat S, Moffat T, Newbold KB. Understanding the healthy immigrant effect in the context of Mental Health challenges: a systematic critical review. J Immigr Minor Health. 2022;24(6):1564–79. 34807354 10.1007/s10903-021-01313-5 PMC8606270

[34] Domnich A, Panatto D, Gasparini R, Amicizia D. The healthy immigrant effect: does it exist in Europe today? Italian Journal of Public Health [Internet]. 2012;9(3). https://ijphjournal.it/article/view/7532.

[35] Helgesson M, Johansson B, Nordquist T, Vingård E, Svartengren M. Healthy migrant effect in the Swedish context: a register-based, longitudinal cohort study. BMJ Open. 2019;9(3):e026972. 30878993 10.1136/bmjopen-2018-026972 PMC6429895

[36] Gong F, Xu J, Fujishiro K, Takeuchi DT. A life course perspective on migration and mental health among Asian immigrants: the role of human agency. Soc Sci Med. 2011;73(11):1618–26. 22019368 10.1016/j.socscimed.2011.09.014

[37] Heise L, Greene ME, Opper N, Stavropoulou M, Harper C, Nascimento M, et al. Gender inequality and restrictive gender norms: framing the challenges to health. Lancet. 2019;393(10189):2440–54. 31155275 10.1016/S0140-6736(19)30652-X

[38] Suárez-Orozco C, Qin DB. Gendered perspectives in psychology: immigrant origin youth. Int Migrat Rev. 2006;40(1):165–98.

[39] Friberg JH, Bjørnset M. Migration, parenting and social control [Internet]. Oslo: Fafo; 2019 [cited 2023 Dec 14]. Report No.: 2019:01. https://www.fafo.no/zoo-publikasjoner/fafo-rapporter/migrasjon-foreldreskap-og-sosial-kontroll.

[40] Bersani BE, Morabito MS. Immigrant disparities in suicide ideation: Variation Across Age of Migration, gender, and Nativity. J Immigr Minor Health. 2020;22(5):881–7. 32162190 10.1007/s10903-020-00993-9

[41] Eurostat. Eurostat - Statistics Explained. 2021 [cited 2023 Dec 14]. Glossary:At-risk-of-poverty rate. https://ec.europa.eu/eurostat/statistics-explained/index.php?title=Glossary:At-risk-of-poverty_rate.

[42] Veling W, Hoek HW, Selten JP, Susser E. Age at migration and future risk of psychotic disorders among immigrants in the Netherlands: a 7-year incidence study. Am J Psychiatry. 2011;168(12):1278–85. 22193672 10.1176/appi.ajp.2011.11010110

[43] Kirkbride JB, Hameed Y, Ioannidis K, Ankireddypalli G, Crane CM, Nasir M, et al. Ethnic minority Status, Age-at-immigration and psychosis risk in rural environments: evidence from the SEPEA Study. Schizophr Bull. 2017;43(6):1251–61. 28521056 10.1093/schbul/sbx010 PMC5737276

[44] Byrow Y, Pajak R, Specker P, Nickerson A. Perceptions of mental health and perceived barriers to mental health help-seeking amongst refugees: a systematic review. Clin Psychol Rev. 2020;75:101812. 31901882 10.1016/j.cpr.2019.101812

[45] Mohammadifirouzeh M, Oh KM, Basnyat I, Gimm G. Factors Associated with Professional Mental help-seeking among U.S. immigrants: a systematic review. J Immigr Minor Health. 2023;1–19. 10.1007/s10903-023-01475-4 PMC10063938 37000385

[46] Nyikavaranda P, Pantelic M, Jones CJ, Paudyal P, Tunks A, Llewellyn CD. Barriers and facilitators to seeking and accessing mental health support in primary care and the community among female migrants in Europe: a feminisms systematic review. Int J Equity Health. 2023;22(1):196. 37752502 10.1186/s12939-023-01990-8 PMC10523615

[47] Shannon PJ, Wieling E, Simmelink-McCleary J, Becher E. Beyond Stigma: barriers to discussing Mental Health in Refugee populations. J Loss Trauma. 2015;20(3):281–96.

[48] Straiton M, Myhre S. Learning to navigate the healthcare system in a new country: a qualitative study. Scand J Prim Health Care. 2017;0(0):1–8. 10.1080/02813432.2017.1397320 PMC5730033 29087232

[49] Orjiako OEY, So D. The role of acculturative stress factors on mental health and help-seeking behavior of sub-saharan African immigrants. Int J Cult Mental Health. 2014;7(3):315–25.

[50] Fjeld-Solberg Ø, Nissen A, Cauley P, Andersen AJ. Mental health and quality of life among refugees from Syria after forced migration to Norway. Oslo: Norwegian Centre for Violence and Traumatic Stress Studies; 2020. p. 71. Report No.: 1/2020.

[51] Hajak VL, Sardana S, Verdeli H, Grimm S. A Systematic Review of Factors Affecting Mental Health and Well-Being of Asylum Seekers and Refugees in Germany. Frontiers in Psychiatry [Internet]. 2021 [cited 2023 Nov 10];12. https://www.frontiersin.org/articles/10.3389/fpsyt.2021.643704. 10.3389/fpsyt.2021.643704 PMC8012840 33815176

[52] Nissen A, Sengoelge M, Solberg Ø. Post-migration stressors and subjective well-being in adult Syrian refugees resettled in Sweden: a gender perspective. Front Public Health. 2021;9:1296. 10.3389/fpubh.2021.717353 PMC8458654 34568258

[53] Rafferty R, Ali N, Galloway M, Kleinshmidt H, Lwin KK, Rezaun M. It affects me as a man: recognising and responding to former refugee men’s experiences of resettlement. An exploratory study in Dunedin, New Zealand. Dunedin: National Centre for Peace and Conflict Studies; 2019. (Policy Paper). Report No.: 2019/1.

[54] Solmi M, Radua J, Olivola M, Croce E, Soardo L, Salazar de Pablo G, et al. Age at onset of mental disorders worldwide: large-scale meta-analysis of 192 epidemiological studies. Mol Psychiatry. 2022;27(1):281–95. 34079068 10.1038/s41380-021-01161-7 PMC8960395

