# Supplementary material for: Age of migration and common mental disorders among migrants in early adulthood: a Norwegian registry study
Source: BMC Psychiatry. 2024 Jul 22;24:521. doi: 10.1186/s12888-024-05963-1 (PMC11265079; doi:10.1186/s12888-024-05963-1)
Supplement: Supplementary file 1 — Supplementary Material 1 [file 12888_2024_5963_MOESM1_ESM.docx]

**Addition file 1: Classification of countries included in each migrant group**

| Migrant group | Number of countries represented | Countries making up around 2/3 of sample* |
| --- | --- | --- |
| Refugees | 163 | Somalia (13.47%), Iraq (11.15%), Eritrea (10.31%), Syria (9.76%), Afghanistan (6.99%), Iran (5.84%), Bosnia Hercegovina (4.75%), Vietnam (4.44%) |
| EEA + ** | 42 | Poland (28.89%), Sweden (13.02%), Lithuania (12.11%), Germany (5.88%), Romania (4.37%), Denmark (4.22%) |
| Non-EEA+ | 168 | The Philippines (14.73%), India (9.32%), Thailand (7,93%), Pakistan (6.2%), Russia (5.9%), China (5.11%), Turkey (3.82%), Brazil (3.07%), Ukraine (2.97%), Serbia (2.58%), Iran (2.03%), Morocco (1.84%), Vietnam (1.69%) |
| *% provided after imputation, **Includes all EEA countries and their associated European territories, North America, Australia and New Zealand, plus smaller countries with close ties to the EEA (e.g. Andorra, Monaco, San Marino) | | |
