# Supplementary material for: Age of migration and common mental disorders among migrants in early adulthood: a Norwegian registry study
Source: BMC Psychiatry. 2024 Jul 22;24:521. doi: 10.1186/s12888-024-05963-1 (PMC11265079; doi:10.1186/s12888-024-05963-1)
Supplement: Supplementary file 2 — Supplementary Material 2 [file 12888_2024_5963_MOESM2_ESM.docx]

**Additional file 2: Imputed of migrant group based on country of origin**

| **Countries** | **Years of migration** | **Recoded as** | **Number recoded** | **Reasons** |
| --- | --- | --- | --- | --- |
| Sweden, Denmark (including Greenland and Faroe Islands), Iceland, Finland | 1969-2019 | EEA+ | 37754 | Nordic citizens have had freedom of movement since the 1950s and have not been required to register a reason for migration upon moving to Norway. |
| Belgium, France, Germany, Italy, Luxemburg, The Netherlands | 1969-2019 | EEA+ | 1265 | Original members of EU |
| UK, Ireland, Greece  Switzerland, Liechtenstein, Austria | 1969-2019 | EEA+ | 881 | Members of EU/EEA, no major political conflicts resulting in (international) fleeing prior to membership^1^ |
| Andorra, San Marino, Monaco, The Vatican City | 1969-2019 | EEA+ | 2 | Associations with the EU, similar history and culture as surrounding / administration countries who have had early EU membership |
| Spain | 1975-2019 | EEA+ | 142 | Member of the EU / post-dictatorship |
| Portugal | 1974-2019 | EEA+ | 54 | Member of the EU / post-dictatorship |
| Czech Republic, Hungary, Latvia, Lithuania, Poland, Malta, Slovakia, Slovenia, Cyprus^2^ | 2004-2019 | EEA+ | 188 | Became member of EU. |
| Bulgaria, Estonia, Romania | 2007-2019 | EEA+ | 103 | Became member of EU |
| Croatia | 2013-2019 | EEA+ | 15 | Became member of EU |
| USA, Canada, Australia, New Zealand | 1969-2019 | EEA+ | 843 | OECD countries; not refugee sending countries (<1% coded as refugee background in original reason for moving) |
| Japan, South Korea^3^ | 1969-2019 | Non-EEA+ | 199 | OECD countries; not refugee sending countries (<1% coded as refugee background in original reason for moving). |
| Mexico | 1994-2019 | Non-EEA+ | 38 | OECD country from 1994; not refugee sending country (<1% coded as refugee background in original reason for moving) |
| Chile | 1973-1989 | Refugees | 1305 | Conflict and dictatorship in Chile resulted in fleeing |
| Vietnam | 1975-1990 | Refugees | 2483 | Vietnam war resulted in fleeing |
| Iran | 1985-1990 | Refugees | 1249 | Political asylum following the revolution and the Iranian-Iraqi war |
| Sri Lanka | 1983-1990 | Refugee | 629 | Civil war from 1983 resulting in fleeing |
| Total |  |  | 47150 |  |

^1^ The Irish Troubles resulted mostly in fleeing to Northern Ireland; ^2^EEA Eastern-European countries were only coded as non-refugee from the year of entry to the EU. This was due to political migration around and following the fall of communism in several of these countries; ^3^Other OECD countries include Israel, Turkey and Chile. We chose not to code impute migrant group for these countries as these are, or have been, refugee sending countries. We did not code Colombia and Costa Rica either, as they became OECD countries in 2020/2021 and the dataset covers up to 2019.

Abbreviations

EU – European Union

EEA – European Economic Area

OECD – The Organisation for Economic Cooperation and Development
