# Supplementary material for: Age of migration and common mental disorders among migrants in early adulthood: a Norwegian registry study
Source: BMC Psychiatry. 2024 Jul 22;24:521. doi: 10.1186/s12888-024-05963-1 (PMC11265079; doi:10.1186/s12888-024-05963-1)
Supplement: Supplementary file 3 — Supplementary Material 3 [file 12888_2024_5963_MOESM3_ESM.docx]

**Additional file 3 – Robustness analysis excluding those missing education level**

| **Odds ratio and 95% confidence intervals for age of migration^1^** | |
| --- | --- |
|  | **OR (95% CI)** |
| Non-migrant | 1 |
| Descendant | 1.08 (1.04-1.12)*** |
| Early childhood | 1.22 (1.16-1.27)*** |
| Late childhood, <19 years | 0.92 (0.87-0.98)** |
| Late childhood migrant, ≥19 years | 1.13 (1.07-1.19)*** |
| Adolescent migrant, <19 years | 0.73 (0.69-0.76)*** |
| Adolescent migrant, ≥19 years | 0.93 (0.86-1.00) |
| Emerging adulthood <19 years | 0.46 (0.45-0.47)*** |
| Emerging adulthood, ≥19 years | 0.81 (0.73-0.90)*** |
| Early adulthood | 0.33 (0.32-0.33)*** |
| Observations=9810724 | |
| N=1778757 | |
| ^1^ adjusted for gender, marital status, education level and low income; **p<0.01; ***p<0.001 | |

| **Odds ratio and 95% confidence intervals for age of migration, migrant group, gender and interactions^1^** | | | | |
| --- | --- | --- | --- | --- |
|  | **Model 1** | **Model 2** | **Model 3** | **Model 4** |
| Early childhood | 2.61 (2.47-2.75)*** | 2.00 (1.87-2.14)*** | 2.54 (2.35-2.74)*** | 1.60 (1.45-1.76)*** |
| Late childhood, <19 years | 2.60 (2.46-2.75)*** | 2.04 (1.90-2.19)*** | 2.65 (2.44-2.88)*** | 1.76 (1.59-1.95)*** |
| Late childhood migrant, 19+ years | 2.21 (2.08-2.34)*** | 1.77 (1.64-1.90)*** | 2.25 (2.07-2.44)*** | 1.49 (1.35-1.64)*** |
| Adolescent migrant, <19 years | 2.05 (1.96-2.15)*** | 1.66 (1.57-1.76)*** | 2.15 (2.02-2.29)*** | 1.47 (1.36-1.59)*** |
| Adolescent migrant, 19+ years | 1.92 (1.77-2.08)*** | 1.57 (1.42-1.72)*** | 2.16 (1.94-2.41)*** | 1.45 (1.28-1.64)*** |
| Emerging adulthood <19 years | 1.38 (1.35-1.41)*** | 1.14 (1.09-1.19)*** | 1.43 (1.37-1.48)*** | 1.17 (1.09-1.24)*** |
| Emerging adulthood, 19+ years | 1.87 (1.67-2.09)*** | 1.43 (1.23-1.68)*** | 2.26 (1.88-2.72)*** | 1.22 (1.24-1.94)*** |
| Early adulthood | 1 | 1 | 1 | 1 |
| Migrant group |  |  |  |  |
| Refugees | 1 | 1 | 1 | 1 |
| EEA+ | 0.66 (0.64-0.68)*** | 0.55 (0.53-0.57)*** | 0.66 (0.65-0.68)*** | 0.38 (0.36-0.40)*** |
| Non-EEA+ | 0.69 (0.67-0.71)*** | 0.57 (0.54-0.59)*** | 0.69 (0.67-0.71)*** | 0.55 (0.52-0.59)*** |
| Women | 2.08 (2.30-2.12)*** | 2.07 (2.03-2.12)*** | 2.13 (2.07-2.20)*** | 1.42 (1.34-1.50)*** |
| Migrant group*age of migration | |  |  |  |
| EEA+*early childhood |  | 1.76 (1.55-1.99)*** |  | 2.50 (2.08-3.01)*** |
| non-EEA+*early childhood |  | 1.78 (1.51-2.11)*** |  | 1.88 (1.46-2.41)*** |
| EEA+*late childhood, <19 years |  | 1.86 (1.59-2.18)*** |  | 2.53 (1.99-3.22)*** |
| non-EEA+*late childhood, <19 years | | 1.53 (1.33-1.76)*** |  | 1.36 (1.09-1.70)*** |
| EEA+*late childhood, 19+ years |  | 1.75 (1.50-2.05)*** |  | 2.46 (1.95-3.10)*** |
| non-EEA+* late childhood, 19+ years | | 1.44 (1.22-1.70)*** |  | 1.38 (1.08-1.77)*** |
| EEA+*adolescence, <19 years |  | 1.75 (1.23-1.64)*** |  | 2.15 (1.77-2.61)*** |
| non-EEA+*adolescence, <19 years |  | 1.37 (1.22-1.54)*** |  | 1.33 (1.12-1.57)*** |
| EEA+*adolescence, 19+ years |  | 1.90 (1.48-2.43)*** |  | 2.76 (1.91-3.99)*** |
| non-EEA+*adolescence, 19+ years |  | 1.28 (1.03-1.60)*** |  | 1.28 (0.93-1.75) |
| EEA+*emerging adulthood, <19 years | | 1.27 (1.20-1.34)*** |  | 1.20 (1.10-1.31)*** |
| non-EEA+*emerging adulthood, <19 years | | 1.33 (1.25-1.42)*** |  | 1.38 (1.24-1.53)*** |
| EEA+*emerging adulthood, 19+ years | | 1.33 (1.00-1.76) |  | 1.46 (0.83-2.59) |
| non-EEA+*emerging adulthood, 19+ years | | 1.77 (1.37-2.30)*** |  | 1.79 (1.10-2.90)* |
| Sex*age of migration | |  |  |  |
| women*early childhood |  |  | 1.06 (0.95-1.17) | 1.57 (1.38-1.79)*** |
| women*late childhood, <19 years |  |  | 0.97 (0.87-1.08) | 1.35 (1.18-1.55)*** |
| women*late childhood, 19+ years |  |  | 0.97 (0.87-1.08) | 1.40 (1.22-1.60)*** |
| women*adolescence, <19 years |  |  | 0.91 (0.84-0.99)* | 1.24 (1.11-1.40)*** |
| women*adolescence, 19+ years |  |  | 0.78 (0.66-0.91)** | 1.10 (0.91-1.34) |
| women*emerging adulthood, <19 years | |  | 0.94 (0.90-0.99)* | 1.00 (0.92-1.09) |
| women*emerging adulthood, 19+ years | |  | 0.75 (0.60-0.94)* | 0.87 (0.64-1.19) |
| Sex*migrant group | |  |  |  |
| women*EEA+ | |  |  | 2.12 (1.97-2.27)*** |
| women*non-EEA+ | |  |  | 1.17 (1.08-1.27)*** |
| Migrant group*age of migration*sex | |  |  |  |
| EEA+*early childhood*woman |  |  |  | 0.49 (0.38-0.63)*** |
| non-EEA+*early childhood*woman | |  |  | 0.81 (0.58-1.13) |
| EEA+*late childhood <19 years*woman | |  |  | 0.53 (0.38-0.73)*** |
| non-EEA+*late childhood <19 years*woman | |  |  | 1.09 (0.82-1.45) |
| EEA+* late childhood, 19+ years*woman | |  |  | 0.51 (0.37-0.69)*** |
| non-EEA+* late childhood, 19+ years*woman | |  |  | 0.96 (0.69-1.33) |
| EEA+*adolescence, <19 years*woman | |  |  | 0.66 (0.51-0.85)** |
| non-EEA+*adolescence, <19 years*woman | |  |  | 0.98 (0.78-1.23) |
| EEA+*adolescence, 19 years+*woman | |  |  | 0.51 (0.31-0.84)** |
| non-EEA+*adolescence, 19 years+*woman | |  |  | 0.95 (0.61-1.48) |
| EEA+*emerging adulthood, <19 years*woman | |  |  | 0.98 (0.87-1.10) |
| non-EEA+*emerging adulthood, <19 years*woman | |  |  | 0.94 (0.82-1.07) |
| EEA+*emerging adulthood, 19 years+*woman | |  |  | 0.81 (0.46-1.57) |
| non-EEA+*emerging adulthood, 19 years+*woman | |  |  | 1.01 (0.57-1.81) |
| Observations=2299300 |  |  |  |  |
| N=426993 |  |  |  |  |
| ^1^ adjusted for gender, marital status, education level and low income; **p<0.01; ***p<0.001 | | | | |
