# Supplementary material for: Age of migration and common mental disorders among migrants in early adulthood: a Norwegian registry study
Source: BMC Psychiatry. 2024 Jul 22;24:521. doi: 10.1186/s12888-024-05963-1 (PMC11265079; doi:10.1186/s12888-024-05963-1)
Supplement: Supplementary file 4 — Supplementary Material 4 [file 12888_2024_5963_MOESM4_ESM.docx]

**Additional file 4: Robustness analysis excluding those missing reason for migration**

| **Odds ratio and 95% confidence intervals for age of migration, migrant group, gender and interactions^1^** | | | | |
| --- | --- | --- | --- | --- |
|  | **Model 1** | **Model 2** | **Model 3** | **Model 4** |
| Early childhood | 2.11 (1.98-2.25)*** | 2.13 (1.97-2.31)*** | 2.91 (2.64-3.19)*** | 1.76 (1.57-1.97)*** |
| Late childhood, <19 years | 1.93 (1.83-2.05)*** | 2.00 (1.87-2.15)*** | 2.69 (2.47-2.92)*** | 1.72 (1.55-1.90)*** |
| Late childhood migrant, ≥19 years | 1.62 (1.51-1.73)*** | 1.72 (1.59-1.86)*** | 2.24 (2.03-2.46)*** | 1.42 (1.27-1.58)*** |
| Adolescent migrant, <19 years | 1.51 (1.45-1.59)*** | 1.62 (1.53-1.72)*** | 2.15 (2.02-2.29)*** | 1.43 (1.32-1.54)*** |
| Adolescent migrant, ≥19 years | 1.37 (1.25-1.49)*** | 1.48 (1.33-1.65)*** | 2.09 (1.85-2.35)*** | 1.34 (1.17-1.54)*** |
| Emerging adulthood <19 years | 1.29 (1.15-1.42)*** | 1.11 (1.06-1.16)*** | 1.42 (1.37-1.47)*** | 1.13 (1.06-1.20)*** |
| Emerging adulthood, ≥19 years | 0.73 (0.72-0.75)*** | 1.27 (1.07-1.50)** | 2.12 (1.74-2.58)*** | 1.33 (1.04-1.70)* |
| Early adulthood | 1 | 1 | 1 | 1 |
| Migrant group |  |  |  |  |
| Refugees | 1 | 1 | 1 | 1 |
| EEA+ | 0.55 (0.54-0.57)*** | 0.45 (0.44-0.47)*** | 0.55 (0.54-0.57)*** | 0.31 (0.29-0.32)*** |
| Non-EEA+ | 0.65 (0.63-0.75)*** | 0.53 (0.51-0.55)*** | 0.65 (0.63-0.67)*** | 0.50 (0.48-0.54)*** |
| Women | 2.03 (1.98-2.07)*** | 2.02 (1.97-2.06)*** | 2.09 (2.03-2.15)*** | 1.37 (1.30-1.45)*** |
| Migrant group*age of migration | |  |  |  |
| EEA+*early childhood |  | 2.46 (2.06-2.93)*** |  | 3.79 (2.91-4.93)*** |
| non-EEA+*early childhood |  | 1.76 (1.48-2.09)*** |  | 1.82 (1.43-2.34)*** |
| EEA+*late childhood, <19 years |  | 2.23 (1.91-2.62)*** |  | 3.10 (2.44-3.94)*** |
| non-EEA+*late childhood, <19 years | | 1.63 (1.42-1.88)*** |  | 1.50 (1.17-1.92)*** |
| EEA+*late childhood, ≥19 years |  | 2.09 (1.72-2.05)*** |  | 3.20 (2.41-4.25)*** |
| non-EEA+* late childhood, ≥19 years | | 1.48 (1.25-1.75)*** |  | 1.50 (1.17-1.92)*** |
| EEA+*adolescence, <19 years |  | 2.02 (1.79-2.30)*** |  | 2.52 (2.07-3.06)*** |
| non-EEA+*adolescence, <19 years |  | 1.45 (1.30-1.62)*** |  | 1.43 (1.21-1.68)*** |
| EEA+*adolescence, ≥19 years |  | 2.08 (1.56-2.79)*** |  | 3.36 (2.19-5.16)*** |
| non-EEA+*adolescence, ≥19 years |  | 1.42 (1.13-1.77)** |  | 1.43 (1.04-1.97)*** |
| EEA+*emerging adulthood, <19 years | | 1.31 (1.24-1.39)*** |  | 1.24 (1.14-1.35)*** |
| non-EEA+*emerging adulthood, <19 years | | 1.36 (1.28-1.44)*** |  | 1.41 (1.27-1.56)*** |
| EEA+*emerging adulthood, ≥19 years | | 1.52 (1.13-2.06)** |  | 2.05 (1.16-3.61)*** |
| non-EEA+*emerging adulthood, ≥19 years | | 1.93 (1.49-2.52)*** |  | 2.07 (1.28-3.37)*** |
| Sex*age of migration | |  |  |  |
| women*early childhood |  |  | 0.98 (0.87-1.11) | 1.47 (1.26-1.71)*** |
| women*late childhood, <19 years |  |  | 0.97 (0.87-1.08) | 1.37 (1.19-1.57)*** |
| women*late childhood, ≥19 years |  |  | 0.97 (0.86-1.10) | 1.44 (1.23-1.68)*** |
| women*adolescence, <19 years |  |  | 0.92 (0.84-1.00) | 1.25 (1.12-1.40)*** |
| women*adolescence, ≥19 years |  |  | 0.79 (0.66-0.94)** | 1.13 (0.92-1.40) |
| women*emerging adulthood, <19 years | |  | 0,94 (0,89-0,98)* | 1.00 (0.92-1.09) |
| women*emerging adulthood, ≥19 years | |  | 0,76 (0,59-0,96)* | 0.92 (0.66-1.29) |
| Sex*migrant group |  |  |  |  |
| women*EEA+ |  |  |  | 2.21 (2.07-2.37)*** |
| women*non-EEA+ |  |  |  | 1.21 (1.12-1.30)*** |
| Sex*migrant group*age of migration | |  |  |  |
| EEA+* early childhood*woman | |  |  | 0.43 (0.30-0.61)*** |
| non-EEA+*early childhood*woman | |  |  | 0.86 (0.61-1.22) |
| EEA+* late childhood <19 years*woman | |  |  | 0.51 (0.38-0.70)*** |
| non-EEA+*late childhood <19 years*woman | |  |  | 1.05 (0.79-1.39) |
| EEA+* late childhood, ≥19 years *woman | |  |  | 0.43 (0.30-0.64)** |
| non-EEA+* late childhood, ≥19 years*woman | |  |  | 0.89 (0.76-1.22) |
| EEA+*adolescence, <19 years*woman | |  |  | 0.64 (0.50-0.83)** |
| non-EEA+*adolescence, <19 years*woman | |  |  | 0.98 (0.78-1.22) |
| EEA+*adolescence, ≥19 years*woman | |  |  | 0.42 (0.24-0.76)** |
| non-EEA+*adolescence, ≥19 years*woman | |  |  | 0.94 (0.60-1.46) |
| EEA+*emerging adulthood, <19 years*woman | |  |  | 0.97 (0.87-1.08) |
| non-EEA+*emerging adulthood, <19 years*woman | |  |  | 0.94 (0.82-1.06) |
| EEA+*emerging adulthood, ≥19 years*woman | |  |  | 0.61 (0.31-1.19) |
| non-EEA+*emerging adulthood, ≥19 years*woman | |  |  | 0.92 (0.51-1.65) |
| Observations=2606363 |  |  |  |  |
| N=522844 |  |  |  |  |
| ^1^ adjusted for gender, marital status, education level and low income; **p<0.01; ***p<0.001 | | | | |
